# PolyPurine Reverse Hoogsteen Hairpins Work as RNA Species for Gene Silencing

**DOI:** 10.3390/ijms221810025

**Published:** 2021-09-16

**Authors:** Eva Aubets, Miguel Chillon, Carlos J. Ciudad, Véronique Noé

**Affiliations:** 1 Department of Biochemistry and Physiology, School of Pharmacy and Food Sciences, Nanoscience and Nanotechnology Institute, IN2UB, University of Barcelona, 08028 Barcelona, Spain; eva395@msn.com (E.A.); cciudad@ub.edu (C.J.C.); 2 ICREA, Institute of Neurosciences at UAB, 08193 Bellaterra, Spain; Miguel.Chillon@uab.es; 3 Vall d’Hebron Institute of Research (VHIR), 08035 Barcelona, Spain

**Keywords:** PPRH, oligonucleotide, survivin, gene targeting, gene silencing, delivery, viral vectors, adenovirus, cancer therapy

## Abstract

PolyPurine Reverse Hoogsteen Hairpins (PPRHs) are gene-silencing DNA-oligonucleotides developed in our laboratory that are formed by two antiparallel polypurine mirror repeat domains bound intramolecularly by Hoogsteen bonds. The aim of this work was to explore the feasibility of using viral vectors to deliver PPRHs as a gene therapy tool. After treatment with synthetic RNA, plasmid transfection, or viral infection targeting the *survivin* gene, viability was determined by the MTT assay, mRNA was determined by RT-qPCR, and protein levels were determined by Western blot. We showed that the RNA-PPRH induced a decrease in cell viability in a dose-dependent manner and an increase in apoptosis in PC-3 and HeLa cells. Both synthetic RNA-PPRH and RNA-PPRH intracellularly generated upon the transfection of a plasmid vector were able to reduce survivin mRNA and protein levels in PC-3 cells. An adenovirus type-5 vector encoding the PPRH against *survivin* was also able to decrease survivin mRNA and protein levels, leading to a reduction in HeLa cell viability. In this work, we demonstrated that PPRHs can also work as RNA species, either chemically synthesized, transcribed from a plasmid construct, or transcribed from viral vectors. Therefore, all these results are the proof of principle that viral vectors could be considered as a delivery system for PPRHs.

## 1. Introduction

In recent years, nucleic acids have proved to be a promising therapy tool for the treatment of a wide range of diseases due to their capacity to modulate specifically any gene of interest [1,2,3,4]. To date, multiple nucleic acids with the aim of inhibiting gene expression have been developed including Triplex Forming Oligonucleotides (TFOs), antisense oligonucleotides (ASOs) [5,6,7,8,9,10,11,12,13,14], small interfering RNAs (siRNAs) [15,16,17,18,19,20,21,22,23,24], microRNAs (mimic and antagomirs) [25,26,27,28,29,30,31,32,33], aptamers [34,35,36,37], ribozymes [38,39], or decoys [40,41,42], some of which obtained regulatory agencies’ approval [43]. In this field, in our laboratory, we developed a new alternative gene-silencing tool called Polypurine Reverse Hoogsteen Hairpins (PPRHs).

PPRHs are single-stranded non-modified DNA hairpins formed by two antiparallel polypurine mirror repeat domains linked by a pentathymidine loop (5T) and bound intramolecularly by Hoogsteen bonds. PPRHs are designed to hybridize to a specific polypyrimidine sequence in the genomic DNA via Watson–Crick bonds, while maintaining its hairpin structure, thus producing a triplex DNA [44]. This triplex structure is possible due the capacity of purines to form Watson–Crick bonds with a pyrimidine and, simultaneously, reverse Hoosgsteen bonds with another purine [45,46]. This triplex conformation leads to the displacement of the polypurine strand of the genomic DNA, resulting in the inhibition of gene expression [44,47]. During the last decade, the ability of PPRHs to downregulate gene expression has been validated using several targets involved in cancer progression both in vitro [47,48,49,50,51,52,53,54,55,56] and in vivo [49]. Furthermore, PPRHs have demonstrated their ability to correct point mutations in the DNA [57,58,59] or to cause exon skipping at the genomic level [60].

In common with other nucleic acids therapeutic tools, the development of safe, efficient, and tissue-specific delivery systems is one of the major translational limitations of PPRHs [61]. Currently, different delivery approaches have been developed that are broadly classified into viral vectors or non-viral vectors [62]. So far, the cationic liposome *N*-[1-(2,3-Dioleoyloxy)propyl]-*N*,*N*,*N*-trimethylammonium methylsulfate (DOTAP) and the cationic polymer Jet-Polyethylenimine (jetPEI) have been used as non-viral vehicles of PPRHs in vitro [50] and in vivo studies [49], respectively. However, some issues regarding their efficiency, toxicity, and specificity encouraged us to search for other alternatives for PPRHs delivery. In this direction, we have recently validated the effectivity of a new cationic gemini surfactant, named DOPY, for the non-viral delivery of PPRHs [63]. Alternatively, in this work, we study viral vectors as carriers for PPRHs.

Viral vectors are replication-deficient viruses genetically modified to delete the disease-causing sequences and to contain the nucleic acid sequence of interest in the viral genome [64]. Several types of viruses for gene therapy have been developed with different properties, including adenoviruses (AdVs) [65], adeno-associated viruses (AAVs) [66], retroviruses [67], lentiviruses [68], herpes simplex-1 viruses (HSV-1) [69], or baculovirus [70].

AdVs are DNA-based viral vectors that present significant advantages: a high transduction efficiency with high levels of transgene expression, the ability to infect both dividing and non-dividing cells, a broad tropism for different cells, lack of host genome integration, large packaging capacities, and the availability of scalable production systems. However, AdVs are highly prevalent in the general population; thus, most people present immunity against one or more human AdV serotypes [64,71]. Positively, these immunogenic properties have been applied for the development of vaccines [72,73] and cancer therapies [74,75]. Therefore, AdV could be suitable candidates for PPRHs as a gene-silencing tool in cancer therapy.

To evaluate viral vectors as a delivery system for PPRHs, we selected a PPRH directed against *survivin* previously validated both in vitro and in vivo (HpsPr-C-WT) [49]. The gene *survivin* (BIRC5) encodes a member of the inhibitor-of-apoptosis proteins (IAP) [76]. Survivin is overexpressed in numerous tumors [77,78,79,80,81,82,83,84] and has been correlated with a more aggressive disease and poor prognosis [78,85,86,87]. In contrast, its levels are undetectable in most normal tissues in adults [78,88,89]. Thus, survivin expression is differentiated between normal and tumor cells. All these characteristics make survivin an ideal target for cancer therapy.

Additionally, since the PPRH would be transcribed in the form of RNA from a viral vector, we first studied whether PPRHs could also work as RNA species. Therefore, we evaluated the effectiveness of a PPRH as an RNA species (synthetic RNA-PPRH) to downregulate survivin. Furthermore, we also checked the effect of the RNA-PPRH intracellularly generated upon the transfection of a plasmid vector.

In this work, we prove that PPRHs can also work not only as DNA species but also as RNA, using RNA chemically synthesized, plasmid, and viral expression vectors. Therefore, we expand the use of viral vectors as a delivery strategy for PPRHs.

## 2. Results

### 2.1. RNA-PPRH Binding Analyses

**Figure 1:**
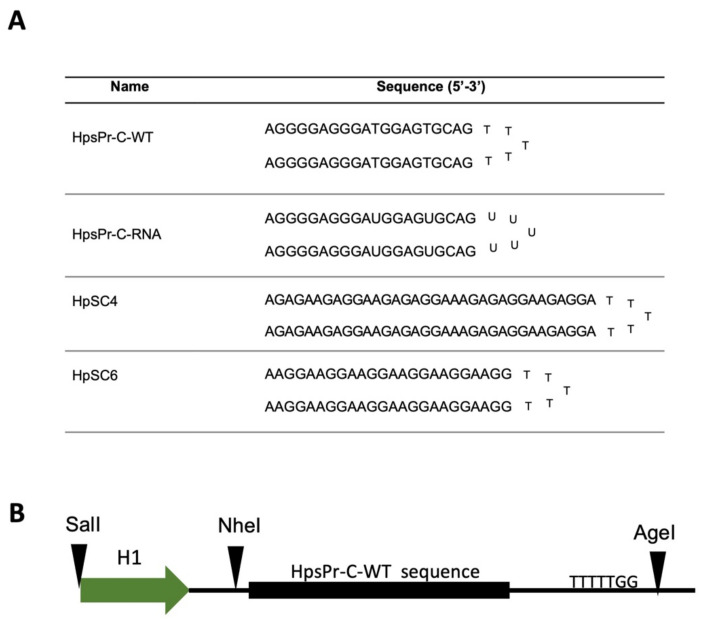
(**A**) Name and sequence of the hairpins and (**B**) schematic representation of the sequence cloned into the cassette. The abbreviations used for the nomenclature of the PPRHs are: Hp—hairpin; Pr—promoter; s—survivin; -C—Coding-PPRH; WT—wild type; RNA—ribonucleotide backbone; SC—scrambled. In the schematic representation of the cassette, the H1 promoter (H1), restriction enzyme sites that allow cloning and five thymidines as a termination signal are indicated.

**Figure 2:**
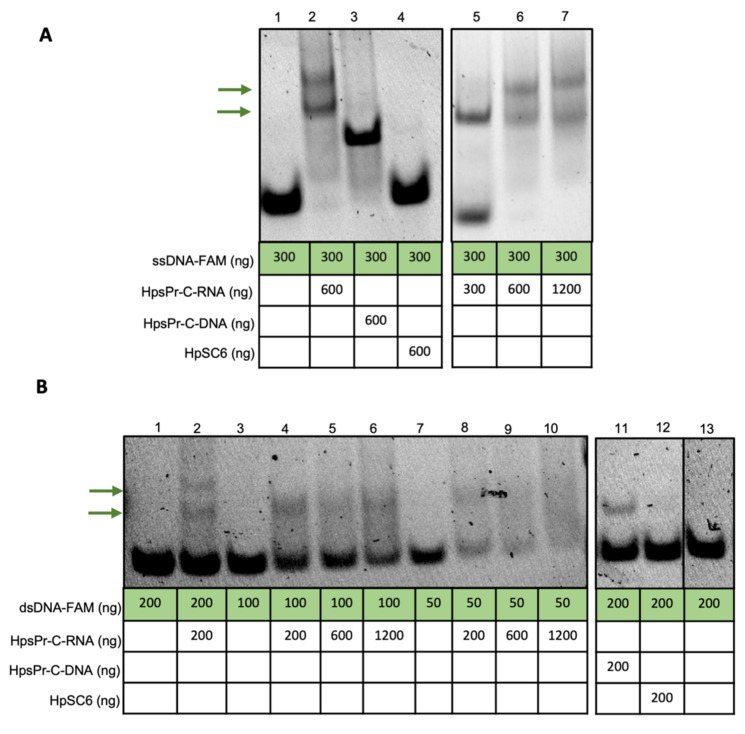
Binding of the RNA-PPRH to its ssDNA or dsDNA target sequence. (**A**) Gel-shift assays using 300 ng of a 5′-FAM labeled ssDNA probe (ssDNA-FAM). The unlabeled oligonucleotides present in each binding reaction are indicated. Lane 1, ssDNA-FAM probe alone; lane 2, ssDNA-FAM plus HpsPr-C-RNA (600 ng); lane 3, ssDNA-FAM plus HpsPr-C-DNA (600 ng); lane 4, ssDNA-FAM plus HpSC6 (600 ng); lane 5, ssDNA-FAM plus HpsPr-C-RNA (300 ng); lane 6, ssDNA-FAM plus HpsPr-C-RNA (600 ng); lane 7, ssDNA-FAM plus HpsPr-C-RNA (1200 ng). (**B**) Gel-shift assays using 200 ng, 100 ng, or 50 ng of a 5′-FAM labeled dsDNA probe (dsDNA-FAM). The unlabeled oligonucleotides present in each binding reaction are indicated. Lane 1, dsDNA-FAM probe alone (200 ng); lane 2, dsDNA-FAM (200 ng) plus HpsPr-C-RNA (200 ng); lane 3, dsDNA-FAM probe alone (100 ng); lane 4, dsDNA-FAM (100 ng) plus HpsPr-C-RNA (200 ng); lane 5, dsDNA-FAM (100 ng) plus HpsPr-C-RNA (600 ng); lane 6, dsDNA-FAM (100 ng) plus HpsPr-C-RNA (1200 ng); lane 7, dsDNA-FAM probe alone (50 ng); lane 8, dsDNA-FAM (50 ng) plus HpsPr-C-RNA (200 ng); lane 9, dsDNA-FAM (50 ng) plus HpsPr-C-RNA (600 ng); lane 10, dsDNA-FAM (50 ng) plus HpsPr-C-RNA (1200 ng); lane 11, dsDNA-FAM (200 ng) plus HpsPr-C-DNA (200 ng); lane 12, dsDNA-FAM (200 ng) plus HpSC6 (200 ng); Lane 13, dsDNA-FAM probe alone (200 ng).

We first tested if the synthetic RNA sequence of HpsPr-C-WT (HpsPr-C-RNA, RNA-PPRH) (Figure 1A) was able to bind to its target single-stranded DNA (ssDNA) or double-stranded DNA (dsDNA). Incubation of increasing amounts of the RNA-PPRH with ssDNA resulted in a progressive disappearance of the band corresponding to the ssDNA probe (Figure 2A, lane 2, 5, 6 and 7) compared to ssDNA alone (Figure 2A, lane 1) and produced two shifted bands, which was probably due to secondary structures in the RNA. Accordingly, the incubation of increasing amounts of the RNA-PPRH with dsDNA also resulted in a progressive disappearance of the band corresponding to the dsDNA probe (Figure 2B, lane 2, 4, 5, 6, 8, 9 and 10) compared to dsDNA alone (Figure 2B, lane 1, 3, 7, and 13) and produced two shifted bands, thus indicating that the RNA-PPRH was able to bind to its target sequence in both ssDNA and dsDNA. Furthermore, no shifted band was originated by the negative control HpSC6 with neither ssDNA (Figure 2A, Lane 4) nor dsDNA (Figure 2B, Lane 12). As a positive control, we incubate the ssDNA and the dsDNA probes with the DNA-PPRH (Figure 2A, lane 3, and Figure 2B, lane 11, respectively).

### 2.2. Effect of a Synthetic RNA-PPRH Targeting Survivin on Viability and Apoptosis

**Figure 3:**
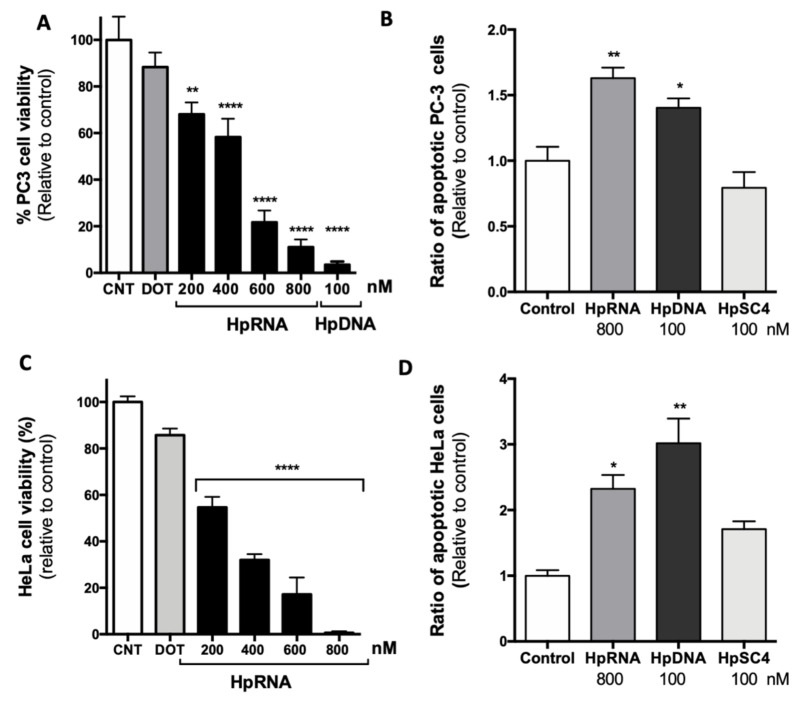
Effect of the RNA-PPRH on viability and apoptosis in PC-3 and HeLa cells. Cell viability assays (10000) were conducted 5 days after transfection. For apoptosis assays, 60000 cells were transfected with 800 nM of the RNA-PPRH HpsPr-C-RNA (HpRNA), 100 nM of the DNA-PPRH HpsPr-C (HpDNA), or 100 nM of a DNA-PPRH negative control HpSC4. Then, 24 h after transfection, cells were collected and processed as specified in an eBioscienceTMAnnexin V Apoptosis Detection kit FITC. The percentage of cells corresponds to Annexin V-positive/IP-negative (early-stage apoptotic cells). (**A**) Effect on viability in PC-3 cells incubated with 15 µM DOTAP (DOT) alone, RNA-PPRH HpsPr-C-RNA (HpRNA, 200–800 nM), or the DNA-PPRH HpsPr-C (HpDNA, 100 nM). (**B**) Effect of RNA-PPRH on apoptosis levels in PC-3 cells. (**C**) Effect on viability in HeLa cells incubated with 15 µM DOTAP (DOT) alone and RNA-PPRH HpsPr-C-RNA (HpRNA, 200–800 nM). (**D**) Effect of RNA-PPRH on apoptosis levels in HeLa cells. Data represent the mean ± SEM from three experiments. Statistical significance was determined using a one-way ANOVA with Dunnett’s multiple comparisons test (* *p* < 0.05, ** *p* < 0.01, **** *p* < 0.0001).

Our previous results demonstrated that the suppression of the antiapoptotic *survivin* gene using HpsPr-C-WT resulted in a decrease in cell survival and an increase in apoptosis in prostate cancer cells [49]. Therefore, the effect of the RNA-PPRH on cell viability and apoptosis was tested in PC-3 cells. The transfection of HpsPr-C-RNA reduced PC-3 cell viability in a dose-dependent manner, with a decrease of 89% relative to control at 800 nM (Figure 3A). As previously reported, 100 nM of HpsPr-C-WT (DNA-PPRH) decreased PC-3 cell viability more than 95%. Furthermore, 24 h after transfection, both HpsPr-C-WT-DNA and HpsPr-C-RNA led to an increase in apoptosis levels in PC-3 cells of 1.4-fold and 1.6-fold, respectively (Figure 3B). In contrast, cells treated with the negative control HpSC4 did not show any increment in apoptosis.

The effect of the RNA-PPRH in viability and apoptosis levels was also tested in HeLa cells. Similar to PC-3 cells, the transfection of HpsPr-C-RNA in HeLa cells led to a decrease in viability in a dose-dependent manner (Figure 3C). Moreover, HeLa cells treated with HpsPr-C-WT-DNA or HpsPr-C-RNA also showed a 2.3-fold or 3-fold increase in apoptosis, respectively (Figure 3D), while the negative control HpSC4 did not produce an increment in the apoptotic cell population.

### 2.3. Effect on the Levels of Survivin mRNA and Protein upon RNA-PPRH Transfection

**Figure 4:**
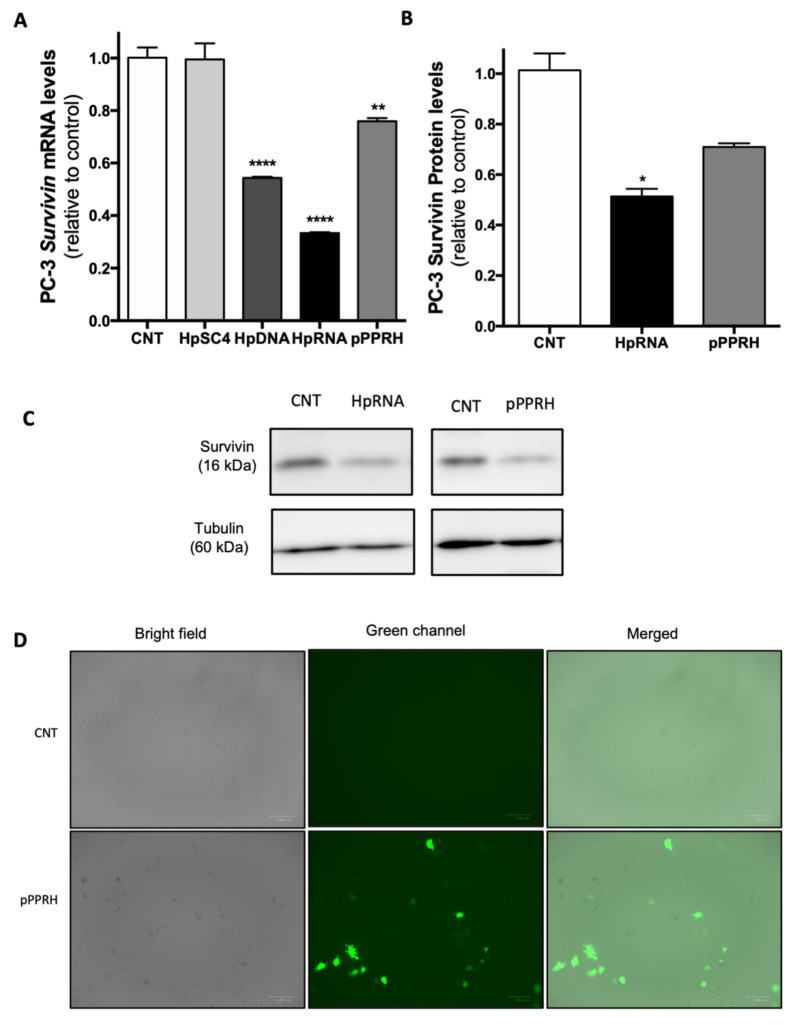
Effect of RNA-PPRH on survivin mRNA and protein levels in PC-3 cells. (**A**) Effect of RNA-PPRH on survivin mRNA levels. PC-3 cells (30000) were transfected with 100 nM of a DNA-PPRH negative control HpSC4, 100 nM of the DNA-PPRH HpsPr-C (HpDNA), 800 nM of HpsPr-C-RNA (HpRNA), or 5 µg of pPPRH-HpsPr-C (pPPRH). Then, 24 h after transfection, or 48 h in the case of pPPRH, survivin mRNA levels were determined by RT-qPCR. TATA-binding protein (TBP) was used to normalize the results. (**B**) Effect of RNA-PPRH on survivin protein levels. PC-3 cells (30000) were treated 24 h with 800 nM of the specific RNA-PPRH HpsPr-C (HpRNA) or 48 h with 5 µg of pPPRH-HpsPr-C (pPPRH); then, protein extracts were obtained to analyze survivin protein levels. (**C**) Representative images of Western blots. Tubulin protein levels were used to normalize the results. (**D**) Fluorescence microscopy images of each condition were taken before analysis. Data represent the mean ± SEM from three experiments. Statistical significance was determined using a one-way ANOVA with Tukey’s multiple comparisons test (* *p* < 0.05, ** *p* < 0.01, **** *p* < 0.0001).

To confirm that the decrease in cell viability and the increase in apoptosis were due to the inhibition of *survivin*, we analyzed its mRNA and protein levels. In terms of *survivin* mRNA levels (Figure 4A), cells treated with HpsPr-C-RNA showed a decrease of nearly 70% relative to the control. Moreover, the positive control HpsPr-C-DNA also reduced *survivin* mRNA levels around 56%, whereas the scrambled negative control did not show a reduction in mRNA levels in PC-3 cells. In the case of survivin protein levels (Figure 4B,C), PC-3 cells transfected with HpsPr-C-RNA showed a decrease of 50%. Therefore, we showed that there is a correlation between the decrease in survivin expression and the decrease in cell viability at 800 nM. Once we verified the effect of the synthetic RNA-PPRH, we also tested the effect of the PPRH when transcribed from a plasmid on PC-3 mRNA (Figure 4A) and protein levels (Figure 4B,C). Five µg of pPPRH-HpsPr-C reduced mRNA levels by 25% and protein levels by 30% relative to the control. Fluorescence microscopy images of cells were taken to verify pPPRH-HpsPr-C expression (Figure 4D).

### 2.4. Cell Viability Assays with AdV Vectors

**Figure 5:**
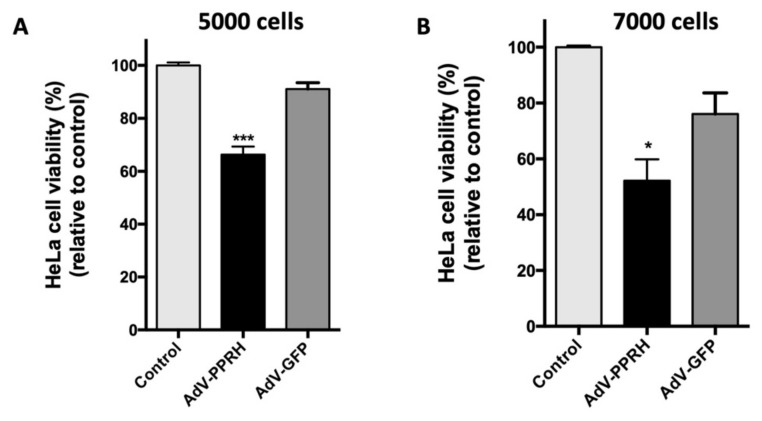
Effect of AdV-PPRH on HeLa cells viability. In total, 5000 (**A**) and 7000 (**B**) cells were plated the day before infection (100 MOI). Error bars represent the standard error of the mean of three experiments. Statistical significance was calculated using one-way ANOVA with Tukey’s multiple comparisons test (* *p* < 0.05, *** *p* < 0.001).

Once we verified that RNA-PPRHs induced survivin silencing, we tested the biological response to the infection of an AdV type-5 vector (AdV5) encoding the PPRH against *survivin* HpsPr-C-WT under the control of the H1 promoter (AdV-PPRH). We started transducing PC-3 cells; however, AdV-PPRH did not induce a significant decrease in viability (data not shown), which was probably due to the low transduction efficiency. For that reason, we switched to using Hela cells. After 72 h of AdV-PPRH transduction, viability assays conducted with this cell line showed a reduction of 34% and 48%, starting with 5000 or 7000 cells, respectively (Figure 5A,B). Moreover, no significant decrease in viability was observed in cells infected with the negative control AdV-GFP, which was an adenovirus encoding GFP.

### 2.5. Effect of AdV-PPRH Infection on Survivin mRNA and Protein Levels

**Figure 6:**
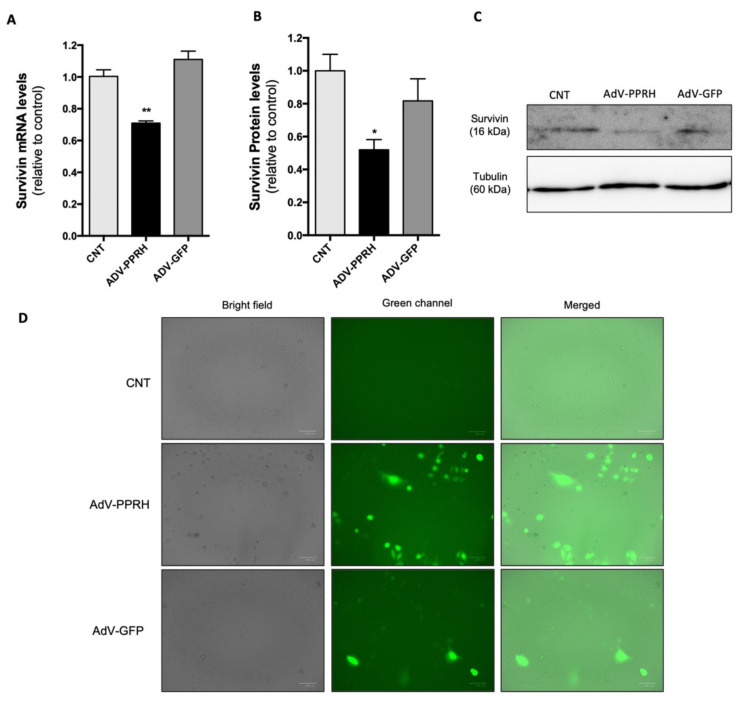
Effect of Adv-PPRH on survivin mRNA and protein levels in HeLa cells. HeLa cells (15,000) were plated the day before infection (100 MOI). mRNA levels (**A**) and protein levels (**B**) were analyzed 72 h after infection. Survivin mRNA levels were determined by RT-qPCR. TATA-binding protein (TBP) was used to normalize the results. (**C**) Representative images of Western blots are shown. Tubulin protein levels were used to normalize the results. (**D**) Fluorescence microscopy images of each condition were taken before each analysis. Error bars represent the standard error of the mean of three experiments. Statistical significance was calculated using one-way ANOVA with Dunnett’s multiple comparisons test (* *p* < 0.05, ** *p* < 0.01). Abbreviation: CNT—control.

To confirm that the decrease in HeLa cell viability was the result of a specific inhibition of *survivin* expression caused by AdV-PPRH, we analyzed the levels of both *survivin* mRNA and protein in HeLa cells 72 h after infection (Figure 6). HeLa cells infected with AdV-PPRH showed a decrease in *survivin* mRNA levels of 30% relative to controls (Figure 6A), whereas the negative control AdV-GFP did not affect mRNA levels. Furthermore, survivin protein levels in HeLa cells infected with AdV-PPRH were reduced by 50% compared to the control (Figure 6B,C), while its levels in cells treated with the negative control AdV-GFP were unaltered in comparison with the control. The infection of cells was monitored through GFP expression using a ZOE Fluorescent Cell Imager (Bio-Rad Laboratories, Inc., Spain). Cell images acquired just before RNA or protein expression analyses are shown in Figure 6D.

## 3. Discussion

The aim of this work was to study the possibility of using viral vectors as a delivery system for PPRHs. Nevertheless, beforehand, we had to determine the molecular effect produced by RNA-PPRHs using RNA chemically synthesized or a plasmid encoding the RNA-PPRH against *survivin*. During the last decade, our laboratory has studied the potential advantages of PPRHs as a gene-silencing tool compared to other nucleic acids [90,91]. PPRHs are more stable than siRNAs, which is probably due to their backbone composed by deoxynucleotides and their hairpin structure [92]. Furthermore, PPRHs exert a more potent effect compared to ASOs [47], and they bind with higher affinity to the target dsDNA than TFOs [51]. In terms of safety, PPRHs are less immunogenic than siRNAs [92], and they do not present nephrotoxicity or hepatotoxicity in vitro [93]. Moreover, pharmacogenomic studies demonstrated the specificity of an PPRH against the *survivin* gene and the lack of off-target effects of an unspecific hairpin [93].

A major objective in our laboratory is to find vehicles to achieve an efficient release of PPRHs at the intracellular and nuclear level so that they can exert their function on their target. The use of viruses for nucleic acids delivery has long been studied, leading to the development of several types of viral vectors and the approval of some of them [64]. In the case of adenoviral vectors, Gendicine and Oncorine [94,95] were the first gene therapies approved for cancer in China. Later on, different vaccines based on adenovirus vectors were approved against Ebola [96] or the global coronavirus disease 2019 (COVID-19) pandemic [97]. This landscape encouraged us to evaluate the use of AdV as a carrier for PPRHs.

Given that the active PPRH sequence would be originated upon transcription from the genome of the AdV vector, we evaluated the effect of a PPRH against *survivin* [49] made out of non-modified ribonucleotides (RNA-PPRH). Importantly, Hoogsteen bonds can also be established between RNA sequences, and thus, polypurine sequences can form RNA hairpins [98]. The PPRHs silencing effect is based on the formation of a triplex structure, leading to the displacement of one of the strands of genomic DNA [44]. Therefore, we first confirmed that the RNA hairpin directed against *survivin* was also able to bind to its target sequence as ssDNA and dsDNA, and thus, it was able to form a triplex structure. Other studies have also corroborated the ability of RNA polynucleotides to recognize DNA duplexes and form triplexes [99]. We also demonstrated that the incubation of this RNA-PPRH in cancer cells leads to a decrease in viability and an increase in apoptosis, although higher concentrations of RNA-PPRH are required compared with those of a DNA-PPRH, which could suggest that DNA-PPRHs are more efficient than RNA-PPRHs. Moreover, we also confirmed that both the RNA-PPRH chemically synthesized and the RNA-PPRH transcribed from a plasmid were able to reduce survivin mRNA or protein levels. In this direction, other groups have also described DNA vectors as a system for the expression of siRNAs to suppress gene expression in mammalian cells [100,101].

Several groups have developed viral vectors with the ability to efficiently deliver therapeutic nucleic acids into mammalian cells, including siRNAs or antisense oligonucleotides [102,103,104,105]. Following this approach, we validated the use of an adenoviral vector as a delivery system for PPRHs in vitro. We demonstrated that the transduction of an AdV5 encoding the PPRH against survivin in cancer HeLa cells induced a reduction in cell viability. Moreover, we showed that this AdV-PPRH was able to effectively downregulate survivin mRNA and protein levels, thus proving the effectivity of viral vectors as a delivery system for PPRHs. Therefore, we demonstrated that PPRH can work using the three RNA-PPRH approaches studied in this work. Comparing them would be a challenging task due to the different methodologies of transfection used and the times of expression in each case. Nevertheless, the main purpose of this work was to validate adenoviral vectors as a putative system for PPRH delivery, not to compare the RNA-PPRH strategies. Although in this work we showed positive results, more work is needed to improve the delivery of these hairpins mediated by viruses.

An important obstacle of adenovirus vectors is their immunogenicity, which was clearly evidenced when the treatment with an adenovirus vector led to the death of an ornithine transcarbamylase-deficient patient [106]. Since then, different strategies to overcome safety problems and to improve the capacity of the transgene size or the durability of transgene expression have been analyzed, such as the removal of different genes of the adenoviral genome to achieve different levels of attenuation [65]. Furthermore, non-human AdV vectors have also been developed (canine, bovine, chimpanzee, ovine, porcine), since humans do not have antibodies against those vectors [64,107,108,109,110].

Different viral vectors are currently being tested in many clinical trials, mostly AdV, AAVs, and retroviruses [64], each of which is characterized by a set of properties. Therefore, the use of viral vectors as a carrier for PPRHs opens many possibilities. For instance, some viral-based systems can achieve long-period expression; thus, viral vectors increase the possibility of reducing the number of administrations of PPRHs. Moreover, proteins of the capsid can be engineered to increase their tissue selectivity [111,112,113,114,115].

Overall, as a conclusion, in this work, we demonstrated that PPRHs can also work as RNA species, either chemically synthesized, transcribed from a plasmid construct, or from viral vectors. Therefore, all these results are the proof of principle that viral vectors could be considered as a delivery system for PPRHs.

## 4. Materials and Methods

### 4.1. Cell Culture

PC-3 prostate cancer cells and HeLa cervix cancer cells, obtained from the cell bank resources from University of Barcelona, were grown in Ham’s F12 medium supplemented with 10% fetal bovine serum (GIBCO, Invitrogen, Barcelona, Spain) and incubated at 37 °C in a humidified 5% CO_2_ atmosphere. Subculture was performed using 0.05% Trypsin (Merck, Madrid, Spain).

### 4.2. PPRHs Design

To evaluate the effect of RNA-PPRHs, we selected the sequence of a DNA-PPRH directed against *survivin* previously validated in our laboratory both in vitro and in vivo (HpsPr-C-WT) [49]. To find the polypurine stretches that would hybridize to the polypyrimidine track of the target gene, we used the Triplex-forming Oligonucleotide Target Sequence Search software (http://utw10685.utweb.utexas.edu/tfo/ MD Anderson cancer center, The University of Texas, accessed 16 September 2021). BLAST analyses were performed to confirm the specificity of the designed PPRHs. As negative controls, we used two scrambled PPRHs: HpSC4 and HpSC6.

The PPRH sequences were synthetized as non-modified DNA or RNA oligonucleotides by Merck (Haverhill, United Kingdom). DNA hairpins were resuspended in sterile Tris-EDTA buffer (1 mM EDTA and 10 mM HCl-Trishydroxymethyl-amino-methane, pH 8.0) (Merck, Madrid, Spain) and stored at −20 °C, whereas RNA hairpins were resuspended in DEPC H_2_O (diethylpyrocarbonate-treated water) (Merck, Madrid, Spain) and stored at −80 °C until their use. The specific sequences for each of the PPRHs used in this work and the negative controls are shown in Figure 1A.

### 4.3. RNA-PPRH Binding Analyses

The capacity of the modified PPRHs to bind to their target sequence in the *survivin* promoter was analyzed using electrophoretic mobility shift assays (EMSA). The dsDNA probe corresponding to the target sequence of *survivin* was obtained by mixing equal amounts of each single-stranded oligodeoxynucleotide in a 150 mM NaCl solution (Forward strand, 5′-[FAM]CTGCACTCCATCCCTCCCCT-3′; Reverse strand, 5′-AGGGGAGGGATGGAGTGCAG-3′). The forward strand was labeled with FAM (6-Carboxyfluorescein) at its 5′-end. The solution was incubated at 90 °C for 5 min and then allowed to cool down slowly to room temperature (about 1 h). The duplex was resolved in a nondenaturing 20% polyacrylamide gel, visualized using UV shadowing (254 nm), and gel-purified. DNA concentration was determined by measuring its absorbance at 260 nm using a NanoDrop ND-1000 spectrophotometer (ThermoFisher, Barcelona, Spain).

The binding of PPRHs to their target sequence was analyzed using two approaches: (I) by incubation of the PPRHs with a ssDNA probe or (II) by incubation of the PPRH with a dsDNA probe. The ssDNA or the dsDNA probes were incubated with the different modified PPRHs in 20 µL reaction mixtures. In both cases, a buffer containing 10 mM MgCl_2_, 100 mM NaCl, 5% glycerol, 20 units of RNAse inhibitor, and 50 mM HEPES, pH 7.2 in H_2_O DEPC was used (Merck, Madrid, Spain). For ssDNA binding reactions, tRNA was added as an unspecific competitor, while for dsDNA binding reactions, Poly(dI:dC) was used (1:2 ratio for both cases, ng probe: ng unspecific competitor). ssDNA binding reactions were incubated for 30 min at 37 °C, whereas dsDNA binding reactions were incubated for 30 min at 65 °C. HpSC6 was used as a negative control in both cases. The products of the binding reactions were resolved by electrophoresis in nondenaturing 8% polyacrylamide gels (PAGE) containing 10 mM MgCl_2_, 5% glycerol, and 50 mM HEPES, pH 7.2 (Merck, Madrid, Spain) at a fixed voltage of 220 V and 4 °C. The ImageQuant software v5.2 (GE Healthcare, Barcelona, Spain) was used to visualize the results.

### 4.4. Plasmid Vector

To proceed to viral-vector delivery, the PPRH sequence had to be cloned in a viral genome. Therefore, we first designed a construct containing the HpsPr-C-WT sequence under the control of the H1 promoter (pPPRH-HpsPr-C) to evaluate their effectiveness. This construct contains the HpsPr-C sequence flanked by the restriction enzyme sites NheI (5′-end) and AgeI (3′-end), which allowed its cloning into the viral genome. The dsDNA sequence was designed to contain a G for the beginning of transcription (5′-end) and a sequence of termination for the end of transcription (TTTTT) (3′-end) Figure 1B. The construct contains the Ampicillin resistance gene and the Enhanced Green Fluorescence Protein (EGFP) gene. Once the construct was produced, it was sent to Macrogen sequencing services to confirm that the insert was correctly cloned (forward primer: 5′-CCCCCTCCCTATGCAAAAGC-3′).

The following sequence was cloned into the AdV genome: 5′-GTCGACTAATATTTGCATGTAGCTATGTGTTCTGGGAAATCACCATAATGTGAAATGTCTTTGGATTTGGGAATCTTATAAGTTCTGTATGAGACCACGCTAGCGGAGGGGAGGGATGGAGTGCAGTTTTTGACGTGAGGTAGGGAGGGGATTTTTGGTCAAGAGCCAAAAATCCCCTCCCTACCTCACGTCAAAAACTGCACTCCATCCCTCCCCTCTTTTTGGACCGGT-3′. The colors indicate the following: in green, the SalI site; in yellow, the H1 promoter; in gray, the NheI site; red G for the beginning of transcription; in blue, the PPRH; in fuchsia, the stop sequence; and in red, the AgeI site.

### 4.5. Viral Vector Production

The batches of AdV5 containing the PPRH sequence were produced in the viral vector production unit (UPV, autonomous university of Barcelona, UAB, Bellaterra, Spain). The batches of AdV5 were produced transfecting the recombinant adenoviral plasmid in HEK293 cells. The AdV particles were purified by double cesium chloride gradient/gel filtration chromatography. The titration was evaluated by Anti-Ad/Hexon Staining and the quantification of Adenovirus Particles by Spectrophotometry.

### 4.6. Transfection of PPRHs

Cells were plated 24 h before transfection, which consisted of mixing DOTAP (Biontex, München, Germany) with the corresponding amount of the PPRH in serum-free medium up to 200 μL. After 20 min of incubation at room temperature, the mixture was added to the cells in a final volume of 1 mL (full medium). Oligodeoxynucleotides PPRHs (DNA-PPRHs) were transfected with a final concentration of 10 µM DOTAP in the cell culture media, whereas non-modified ribonucleotide PPRHs (RNA-PPRHs) were incubated with 15 µM of DOTAP.

### 4.7. Transfection of Vector

Cells were plated in 6-well dishes one day before transfection. The transfection consisted of incubating FuGENE^®^6 (Promega Biotech Ibérica, S.L., Madrid, Spain) for 5 min in serum-free medium, which was followed by the addition of plasmid DNA (5 µg) and incubation for 15 min. The ratio of FuGENE^®^6 (µL) to DNA plasmid (µg) was 3:1. The final volume for each reaction was 100 µL. Then, the mixture was added to the cells in a final volume of 1 mL (full medium). Plasmid transfection efficiency was monitored through EGFP expression using a ZOE Fluorescent Cell Imager (Bio-Rad Laboratories, Inc., Barcelona, Spain).

### 4.8. Transduction of Human Cells

Cells were plated in 24-well dishes and infected with 100 MOI (multiplicity of infection) in a final volume of 300 µL. Six hours after infection, culture medium was added up to 1000 µL. Viruses infection efficiency was monitored through GFP expression using a ZOE Fluorescent Cell Imager (Bio-Rad Laboratories, Inc., Barcelona, Spain).

### 4.9. Cell Viability Assays

Cells were plated in 6-well dishes (10000) in F12 medium or 24-well dishes (5000 or 7000) in assays conducted with viruses. Five days after transfection, or three after infection in the case of the virus tests, 0.63 mM of 3-(4,5-dimetilthyazol-2-yl)-2,5-dipheniltetrazolium bromide and 100 μM sodium succinate (both from Merck, Madrid, Spain) were added to the culture media and incubated for 2.5 h at 37 °C. After incubation, culture media were removed and the lysis solution (0.57% of acetic acid and 10% of sodium dodecyl sulfate (SDS) in dimethyl sulfoxide) (Merck, Madrid, Spain) was added. Absorbance was measured at 560 nm in a Modulus Microplate spectrophotometer (Turner BioSystems, Madrid, Spain). Cell viability results were expressed as the percentage of cell survival relative to the controls.

### 4.10. Annexin V Apoptosis Detection Kit FITC

Cells (60000) were plated in 24-well dishes in F12 medium. One day after transfection, the levels of apoptosis were analyzed using the eBioscience^TM^Annexin V Apoptosis Detection kit FITC (Thermo Fisher Scientific, Madrid, Spain). Briefly, cells were collected by trypsinization, centrifuged at 800× *g* at 4 °C for 5 min, and washed once in PBS and once in 1× binding buffer from the kit. The pellet was resuspended in 100 µL of 1× binding buffer, and 5 µL of fluorochrome-conjugated Annexin V was added. After 15 min of incubating at room temperature, cells were washed in 1× binding buffer, resuspended in 200 µL of 1× binding buffer, and 5 µL of Propidium Iodide Staining solution was added. Then, flow cytometry analyses were performed in a Gallios flow cytometer (Beckman Coulter, Inc., Barcelona, Spain) at the Techno-Scientific facilities of the University of Barcelona. Annexin V-positive and IP-negative cells were considered as early-stage apoptotic cells, Annexin V-positive and IP-positive cells were considered as late-stage apoptotic and necrotic cells, and Annexin V-negative and IP-negative cells were considered as living cells.

### 4.11. RNA Extraction

Total RNA was extracted from PC-3 and HeLa cells using TRIzol^®^ (Life Technologies, Madrid, Spain) following the manufacturer’s specifications and pooling 3 independently treated wells for each experiment. RNA was quantified by measuring its absorbance at 260 nm using a NanoDrop ND-1000 spectrophotometer (Thermo Scientific, Madrid, Spain).

### 4.12. Reverse Transcription

cDNA was synthesized in a 20 μL reaction mixture containing 1 µg of total RNA, 125 ng of random hexamers (Roche, Barcelona, Spain), 500 μM of each dNTP (Bioline, Barcelona, Spain), 2 μL of buffer (10×), 20 units of RNAse inhibitor, and 200 units of Moloney murine leukemia virus reverse transcriptase (Last three from Lucigen, Wisconsin, USA). The reaction was incubated at 42 °C for 1 h.

### 4.13. Reverse Transcription

A QuantStudio 3 Real-Time PCR System (Applied Biosystems, Barcelona, Spain) was used to conduct these experiments. Survivin (BIRC5) mRNA TaqMan probe (Hs04194392_s1; Life Technologies, Barcelona, Spain) was used to determine *survivin* mRNA levels, and the TATA-binding protein (TBP) mRNA TaqMan probe (Hs00427620_m1, Life Technologies, Barcelona, Spain) was used as the endogenous control. The reaction was conducted in 20 μL containing 1× TaqMan Universal PCR Mastermix (Applied Biosystems, Madrid, Spain), 0.5× TaqMan probe (Applied Biosystems, Madrid, Spain), and 3 μL of cDNA. PCR cycling conditions were 10 min denaturation at 95 °C followed by 40 cycles of 15 s at 95 °C and 1 min at 60 °C. mRNA quantification was performed using the ΔΔCt method, where Ct is the threshold cycle that corresponds to the cycle when the amount of amplified mRNA reaches the fluorescence threshold.

### 4.14. Western Blot Analyses for Survivin Detection

Total protein extracts from PC-3 cells (30000) were obtained 24 h after transfection or from HeLa cells (15000) 72 h after transduction. Extracts were obtained using 100 μL of RIPA buffer (1% Igepal, 0.5% sodium deoxycholate, 0.1% SDS, 150 mM NaCl, 1 mM EDTA, 1 mM PMSF, 10 mM NaF, and 50 mM Tris-HCl, pH 8.0) supplemented with Protease inhibitor cocktail (P8340) (all from Merck, Madrid, Spain) and pooling 3 independently treated wells for each experiment. Extracts were incubated 5 min at 4 °C, and cell debris was removed by centrifugation (16,300× *g* at 4 °C for 10 min).

Whole-protein extracts (100 µg) were electrophoresed in 15% SDS-polyacrylamide gels and transferred to Immobilon-P polyvinylidene difluoride membranes (Merck, Madrid, Spain) using a semidry electroblotting system. Blocking was performed using a 5% skim milk solution. Then, membranes were probed with primary antibodies against survivin (5 µg/mL; AF886, Bio-Techne R&D Systems, S.L.U., Madrid, Spain), or α-Tubulin (1:100 dilution; CP06, Merck, Darmstadt, Germany). Secondary horseradish peroxidase-conjugated antibodies were anti-rabbit (1:2000 dilution; P0399, Dako, Glostrup, Denmark) for survivin and anti-mouse (1:2500 dilution; sc-516102, Santa Cruz Biotechnology, Heidelberg, Germany) for α-tubulin detection. Chemiluminescence was detected with the ImageQuant LAS 4000 mini (GE Healthcare, Barcelona, Spain). Quantification was performed using the ImageQuant 5.2 software.

### 4.15. Statistical Analyses

Statistical analyses were performed using GraphPad Prism 6 (GraphPad Software, California, USA). All data are shown as the mean ± SEM of at least three independent experiments. The levels of statistical significance were denoted as follows: *p* < 0.05 (*), *p* < 0.01 (**), *p* < 0.001 (***), or *p* < 0.0001 (****).
